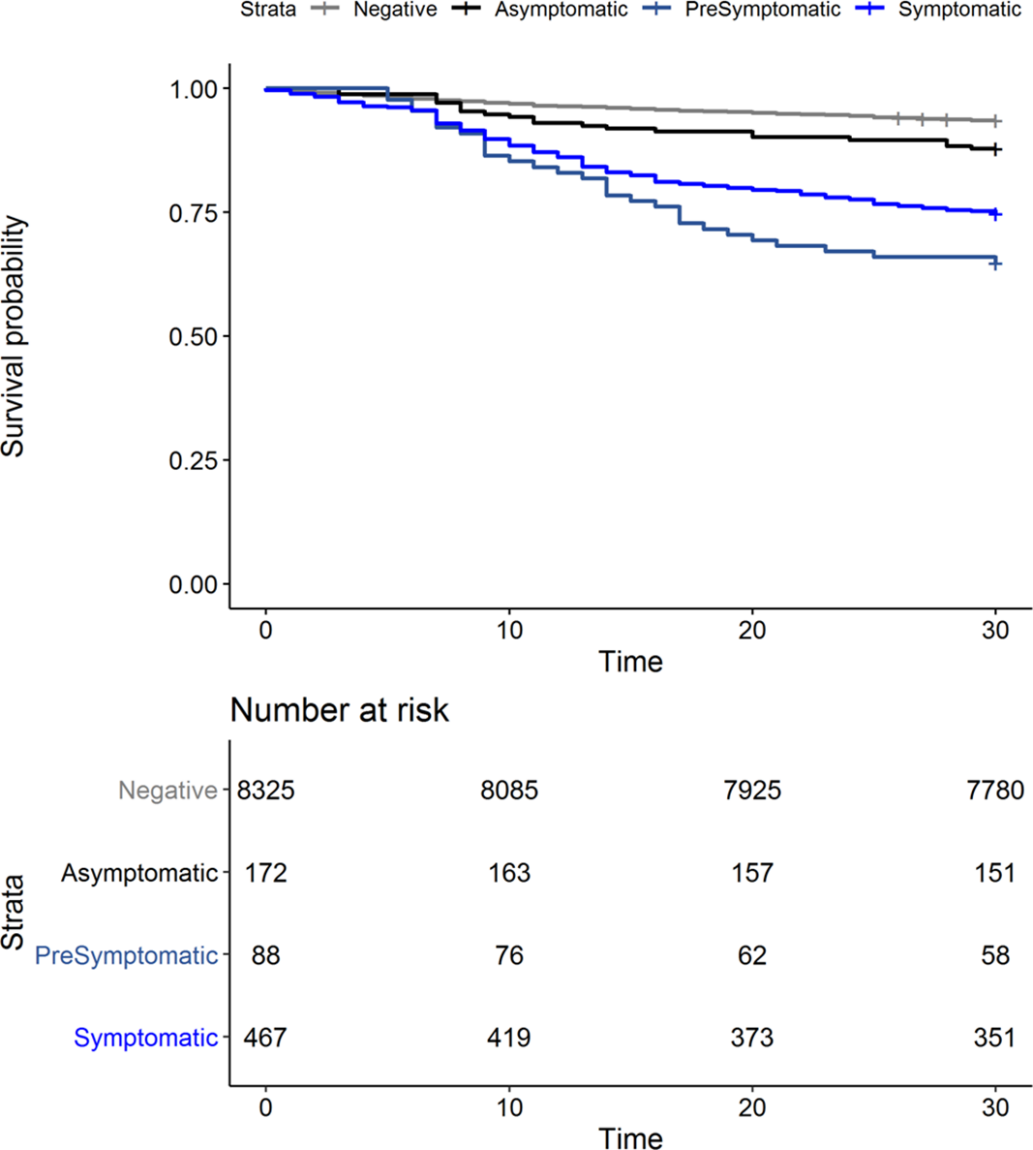# Mortality Among Veterans’ Affairs Community Living Center (CLC) Residents with COVID-19

**DOI:** 10.1017/ash.2021.102

**Published:** 2021-07-29

**Authors:** Taissa Bej, Brigid Wilson, Sunah Song, Janet M Briggs, Richard Banks, Sonya Kothadia, Federico Perez, Robin Jump, Nicole Mongilardi

## Abstract

**Background:** Outcomes among nursing home residents with asymptomatic compared to symptomatic COVID-19 are not well characterized. We assessed all-cause mortality among Veterans’ Affairs (VA) community living center (CLC) residents; we compared those residents with a negative SARS-CoV-2 test to residents with symptomatic, presymptomatic, and asymptomatic SARS-CoV-2 infections. **Methods:** We conducted a national retrospective cohort study of CLC residents tested for COVID-19 between March 1 and July 31, 2020, based on data compiled through the VA COVID-19 shared data resource. Among those with a positive SARS-CoV-2 test, residents were considered symptomatic if they had experienced COVID-19 symptoms in the 30 days prior to the test. Residents were considered presymptomatic if they did not experience symptoms in the 30 days prior to testing and developed a fever (>38°C) or required supplemental oxygen within 14 and 60 days, respectively, following the test. Residents were considered asymptomatic in the absence of these pre- and posttest symptoms. **Results:** From March 1 to July 31, 2020, of 9,052 CLC residents screened for COVID-19, 8,325 (92%) tested negative (Table 1). Among 727 residents with positive tests, 467 (64%) were symptomatic, 88 (12%) were presymptomatic, and 172 (24%) remained asymptomatic. We observed significant differences in the racial makeup of these disease groups. Among CLC residents who were symptomatic or presymptomatic, 176 (32%) of 555 were black compared to 39 (23%) of 172 who were asymptomatic and 1,810 (22%) of 8,325 who tested negative for SAR-CoV-2. All-cause 30-day mortality rates for symptomatic and presymptomatic residents were 25% and 34%, respectively, which exceeded the all-cause 30-day mortality of asymptomatic residents (12%) and residents with a negative test (6%) (Figure 1). **Conclusions:** More than one-third of CLC residents with COVID-19 were asymptomatic at the time of testing. This finding highlights the importance of vigilant infection prevention and control measures. Our finding that mortality among presymptomatic residents exceeded that of symptomatic residents raises consideration for enhancing supportive care measures, such as supplemental oxygen and mitigation of inflammatory reactions, as a means to reduce mortality among nursing home residents with presymptomatic SARS-CoV-2 infections.

**Funding:** No

**Disclosures:** None

**Table 1.**
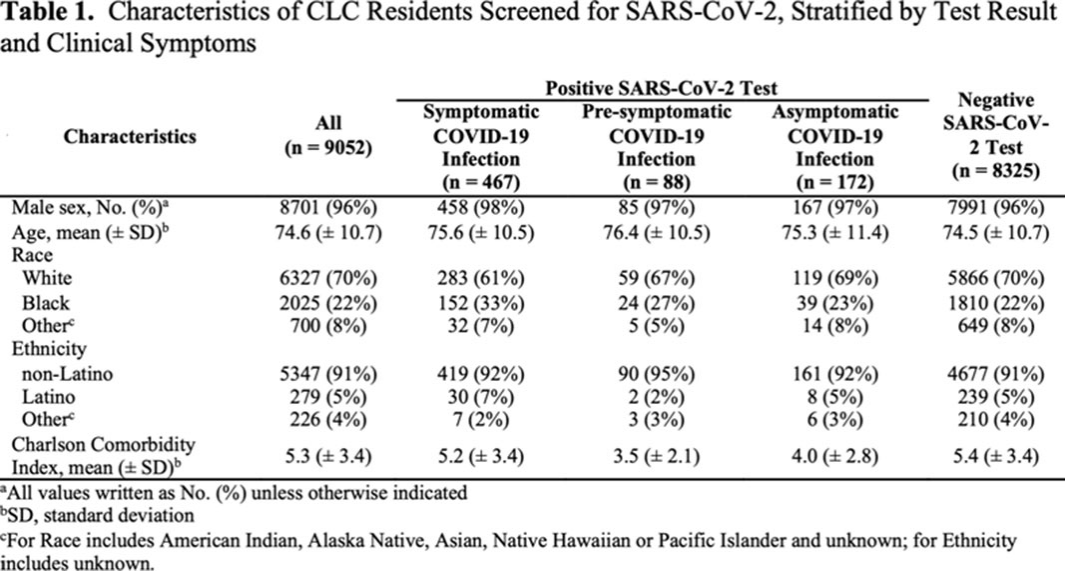

**Figure 1.**